# *Lysimachiacoriacea* (Primulaceae, Myrsinoideae), a new species from Chongqing, China

**DOI:** 10.3897/phytokeys.215.91488

**Published:** 2022-12-16

**Authors:** Hai-Fei Yan, Ya Huang, Hong-Jing Zhang, Si-Rong Yi

**Affiliations:** 1 Key Laboratory of Plant Resources Conservation and Sustainable Utilization, South China Botanical Garden, Chinese Academy of Sciences, Guangzhou 510650, China South China Botanical Garden, Chinese Academy of Sciences Guangzhou China; 2 South China National Botanical Garden, Guangzhou 510650, China South China National Botanical Garden Guangzhou China; 3 Chongqing Three Gorges Medical College, Wanzhou, Chongqing 404120, China Chongqing Three Gorges Medical College Chongqing China

**Keywords:** field expedition, *Lysimachia*, morphology, Nanchuan County, taxonomy

## Abstract

A new species, *Lysimachiacoriacea*, from Chongqing, China, is described and illustrated. It is assigned to subgen. Lysimachiasect.Nummulariaser.Paridiformes and resembles L.paridiformisvar.stenophylla, but is characterised by smaller leathery leaves with black glandular striations near the margin. It is also similar to *L.nanpingensis* in its two to three pairs of leaves sub-whorled at the stem apices, but differs by smaller leathery leaves and densely glandular stem, petiole and pedicel, and calyx lobes with sparse black glandular stripes.

## ﻿Introduction

*Lysimachia* L. is the largest genus in the tribe Lysimachieae (Primulaceae), which consists of approximately 200 species worldwide (Chen and Hu 1979; Chen and Hu 1989; Hu and Kelso 1996; Marr and Bohm 1997; Hao et al. 2004; Julius et al. 2016). Traditionally, the classification of Lysimachieae includes six genera (*Anagallis* L., *Asterolinon* Hoffmanns. & Link, *Glaux* Tourn. ex L., *Lysimachia*, *Pelletiera* A.St.-Hil., and *Trientalis* Ruppius ex L.) based on several characters, such as the dehiscing pattern of the capsule and the number of corolla lobes. Molecular evidence even suggested expanding the delimitation of *Lysimachia* by including all satellite genera in the tribe (e.g. Anderberg et al. 2007). China has been considered as the diversity centre of this genus with approximately 130 species recorded in Flora of China (Hu and Kelso 1996). Subsequently, at least 20 new species have been discovered in the last two decades (Peng and Hu 1999; Shao et al. 2004; Shao et al. 2006; Yan and Hao 2012; Liu et al. 2014a, b; Estes et al. 2015; Zhou et al. 2015; Baskose et al. 2016; Julius et al. 2016; Wang et al. 2016; Yan et al. 2017; Wang et al. 2018; Huang et al. 2019; Liu et al. 2020; Mou et al. 2020; Yi 2020; Ju et al. 2021; Lu et al. 2021). In recent years, multiple field expeditions have been conducted in the Municipality of Chongqing, China. During several fieldworks, a species new to science of *Lysimachia* was discovered and is described below.

## ﻿Materials and methods

Multiple field investigations were conducted between April 2019 and July 2022 to collect the specimens of the putative new species. The morphological descriptions are based on both living and dried materials, which are deposited in the Herbaria of South China Botanical Garden, Chinese Academy of Sciences (IBSC) and Kunming Institute of Botany, Chinese Academy of Sciences (KUN). This study was based on an examination of herbarium specimens at IBSC, PE, KUN and digital images from the Chinese Virtual Herbarium (CVH: https://www.cvh.ac.cn/index.php), Global Plants JSTOR (http://plants.jstor.org/) and Plants of the World Online (POWO: http://www.plantsoftheworldonline.org/). In addition, the descriptions of its most similar species (L.paridiformisvar.stenophylla Franch. and *L.nanpingensis* F.H. Chen & C.M. Hu) from the relevant taxonomic literature (Chen and Hu 1979; Chen and Hu 1989; Hu and Kelso 1996) were also consulted. The conservation assessment of the putative new species was undertaken using IUCN categories of threat (IUCN Standards and Petitions Committee 2022).

## ﻿Taxonomic treatment

### ﻿ Lysimachia coriacea

Taxon classificationPlantaeEricalesPrimulaceae

S.R. Yi & H.F. Yan sp. nov.

65CA2A69-03B7-5794-AA75-AC26634853EB

urn:lsid:ipni.org:names:77310050-1

Figs 1 , 2

#### Type.

China. Chongqing: Nanchuan District, Nanping Town, Shangenqiao, 29°02'N, 107°08'E, 740 m a.s.l., 28 April 2019, *Si-Rong Yi YSR8174* (holotype IBSC!; isotypes KUN!).

#### Diagnosis.

The new species belongs to subgen. Lysimachiasect.Nummulariaser.Paridiformes Chen & Hu (1979: 36) characterized by verticillate upper leaves, scale-like lower leaves and umbellate inflorescence. It is similar to L.paridiformisvar.stenophylla in having scale-like lower leaves, upper leaves in a terminal whorl and terminal umbels, but it is easily distinguished by its smaller and leathery leaves (vs. papery) only with black glandular stripes near the margin (vs. black glandular stripes on the whole leaf blade), pedicels with dense stalked glands (vs. glabrous), and corollas without black glandular stripes (vs. occasionally with black glandular stripes) (Hu and Kelso 1996). At first glance, it also looks similar to *L.nanpingensis*, but differs by its smaller leathery leaves (vs. papery) and densely glandular stem, petiole and pedicel (vs. densely fulvous pubescent), and glabrous calyx lobes with sparse black glandular stripes (vs. pubescent and sparsely reddish glandular punctate). A more detailed comparison of the three species is provided in Table 1.

**Figure 1.**
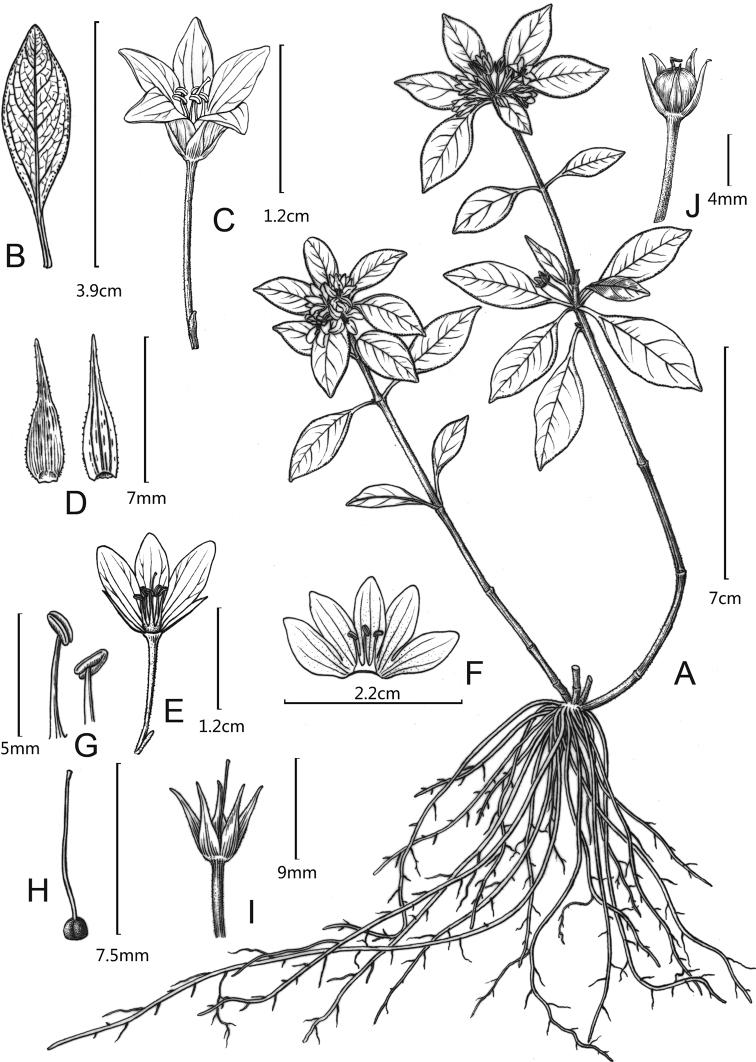
*Lysimachiacoriacea***A** habit **B** leaf **C** flower **D** calyx lobes, abaxial view (right) and adaxial view (left) **E, F** opened corolla, showing filaments connate at base **G** anthers, front view (left) and back view (right) **H** pistil **I** flower with corolla removed **J** young fruit with persistent calyx. Drawn by Yun-Xiao Liu.

#### Description.

Herbs perennial, 5–15 cm tall. ***Rootstock*** with numerous fibrous roots. ***Stem*** terete, erect or lower part procumbent, rooting at nodes, simple or branched, with dense stalked glands when young. ***Leaves*** opposite, lowest 1–2 pairs scale-like, upper 2–3 pairs closely crowded; blades elliptic or ovate-elliptic, 1.8–3.5 × 1.0–1.8 cm, leathery, adaxially dark green, smooth and glabrous, lustrous; abaxially light green, glabrous, black glandular striate near entire margin, base cuneate, apex acute, mid-vein impressed above, raised below, lateral veins and veinlets inconspicuous; petiole 5–15 mm long, with dense stalked glands. ***Flowers*** 1–5, crowded at apex of stems, bracts oblanceolate, 6 mm long, green, sparsely glandular; pedicel 6–15 mm long, with dense stalked glands, erect in fruit; calyx lobes narrowly lanceolate, 4.5–6.8 × 0.9–1.5 mm, divided to base, apex acuminate, abaxially sparsely black glandular striate, margin glandular, membranous; corolla yellow, tube 2–3 mm high, lobes elliptic, 6–9 × 5.5–6.8 mm, apex obtuse or slightly emarginate; filaments connate basally into a tube, ca. 2 mm high, with dense granular glandular spots, free parts 2–3 mm long; anthers oblong, 0.8–1.2 mm long, dorsifixed; ovary subglobose, 0.9–1.2 mm in diam., glabrous; style 4.5–6.0 mm long, glabrous. ***Capsule*** subglobose, 2.5–4.5 mm in diam.

**Figure 2.**
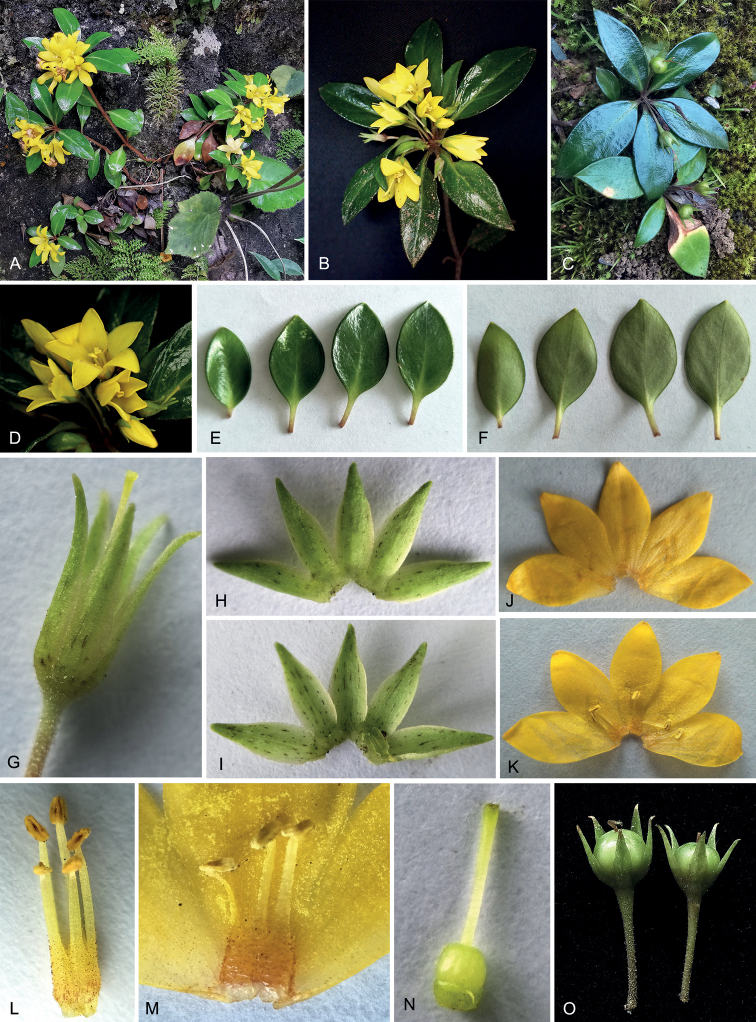
*Lysimachiacoriacea***A** flowering plants **B** inflorescence **C** fruiting plants **D** flowers **E, F** leaves **G** flower with corolla removed **H** calyx, abaxial view **I** calyx, adaxial view **J** corolla, abaxial view **K** corolla, adaxial view **L–M** stamens **N** pistil **O** young fruits. Photographed by Si-Rong Yi.

#### Distribution and habitat.

*Lysimachiacoriacea* is only known from the western slopes of Jinfo Mountain, Nanchuan, Chongqing, China. It grows on damp rocks or cliffs in forests, along roadsides and in mountain valleys at elevations of 740–1,300 m.

#### Conservation status.

This new species has only been found on Jinfo Mountain with at least four populations, where its area of occupancy is less than 10 km^2^. Fortunately, the current distribution area of the species is under the protection of the Jinfo Mountain National Natural Reserve. Thus, it is assigned the status of “Least Concern” (LC) according to the IUCN Red List Categories and Criteria (IUCN Standards and Petitions Committee 2022).

#### Phenology.

Flowering from April to May and fruiting from May to June.

#### Etymology.

Latin *coriacea*, leathery, alluding to texture of leaves.

#### Chinese name.

革叶过路黄 (Gé Yè Guò Lù Huáng).

#### Additional specimens examined.

**China. Chongqing**: Nanchuan District, Nanping Town, Shangenqiao, 29°02'N, 107°08'E, 740 m a.s.l., in shady fissures of wet rocks, 7 June 2019, *Si-Rong Yi YSR8004* (KUN1510469!); Nanchuan District, Nanping Town, Huangniya, on a damp rocky roadside, 1,300 m a.s.l., 28 May 1986, *Z. Y. Liu 0663* (PE01895529!); Nanchuan District, Dutouma (from Lanba to Sanhui), on the roadside, 860 m a.s.l., 7 May 1957, *J.H. Xiong & Z.L. Zhou* 90705 (IBSC0019656!); Nanchuan District, Sanhui Dianchanggou, on a damp rock in forests, 970 m a.s.l., 5 July 1957, *J.H. Xiong & Z.L. Zhou* 91820 (IBSC0019657!).

**Table 1.** Comparison of diagnostic characters of *Lysimachiacoriacea*, L.paridiformisvar.stenophylla and *L.nanpingensis*.

	*L.coriacea*	L.paridiformisvar.stenophylla	*L.nanpingensis*
Stem	with dense stalked glands when young	glabrous	with dense fulvous multicellular hairs
Leaf blade	elliptic or ovate-elliptic, 1.8–3.5 × 1.0–1.8 cm, leathery; base cuneate, apex acute; with black glandular stripes near the margin; veins inconspicuous, except mid-vein	narrowly elliptic to broadly lanceolate or linear-lanceolate, 4–16 × 1.2–5 cm, papery; base cuneate, apex short acuminate; with or without black glandular stripes; veins 4 or 5 pairs, conspicuous	elliptic to ovate-elliptic, 3.5–5.5 × 2–4.5 cm, papery; base subrounded, apex acute; indistinctly glandular punctate; veins inconspicuous, except mid-vein
Petiole	5–15 mm long; with dense stalked glands	sessile or subsessile; glabrous	3–12 mm long; densely fulvous pubescent
Calyx lobes	4.5–6.8 × 0.9–1.5 mm; glabrous on both sides, sparsely ciliate on the margin, with sparse black glandular stripes	8–13 × 2.5–3.5 mm; glabrous on both sides, occasionally ciliate on the margin, occasionally with black glandular stripes	6–7.5 × 1.2–1.9 mm; pubescent on the abaxial surface, obscurely glandular punctate
Pedicel	6–15 mm long, erect in fruit, with dense stalked glands	3–15 mm long; erect in fruit, glabrous	4–9 mm long, recurved in fruit, densely fulvous pubescent
Corolla lobes	6–9 × 5.5–6.8 mm, without glandular stripes	9–11 × 4–4.5 mm, with or without black glandular stripes	9–11 × 3.5–4 mm, sparsely reddish glandular punctate

#### Notes.

Specimens of the new species were first collected by Ji-Hua Xiong and Zi-Lin Zhou in 1957 and deposited in IBSC (IBSC0019656 and IBSC0019657). The two specimens were identified as “Lysimachiaparidiformisvar.stenophylla Franch.” by Chi-Ming Hu. However, Hu should also notice the differences between this specific taxon and L.paridiformisvar.stenophylla, because he wrote a temporary name “*Lysimachianitida* Chen et C.M. Hu” on the annotation labels of the specimens. Later, specimens of the new species were collected by some other collectors (see above).

## Supplementary Material

XML Treatment for Lysimachia coriacea
